# Development and Application of InDel Markers Linked to Fruit-Shape and Peel-Colour Genes in Wax Gourd

**DOI:** 10.3390/genes13091567

**Published:** 2022-08-31

**Authors:** Xiaochun Huang, Wenting Wu, Liwen Su, Haixuan Lv, Zhikui Cheng, Wenrui Yang, Lifeng Nong, Ting Liu, Yong Chen, Peng Wang, Zhengguo Liu

**Affiliations:** 1 College of Agricultural, Guangxi University, Nanning 530004, China; 2 Institute for New Rural Development, Guangxi University, Nanning 530004, China; 3 Institute of Vegetable Research, Guangxi Academy of Agricultural Sciences, Nanning 530004, China

**Keywords:** fruit shape, gene, InDel marker, peel colour, wax gourd

## Abstract

The wax gourd is commonly grown in many countries because of its high nutritional and economic value. While the genes for the fruit shape and peel colour of wax gourd have been reported, the InDel markers linked to these genes remain undeveloped. In this study, the InDel markers linked to fruit-shape (*Bch02G016830*) and peel-colour (*Bch05G003950*) genes were developed from resequenced data. We used 120 inbred lines, 536 isolated populations, and 4 commercial hybrids to evaluate the validity and application value of the InDel markers. The accuracy rates of nine pairs of fruit-shape InDel markers (GX1-GX9) were 84.16–91.66% in 120 inbred lines. The accuracy rates of 27 pairs of peel-colour InDel markers (PS1-PS27) within approximately 3.0 Mb upstream and 3.0 Mb downstream of the peel-colour gene were 100% and those of 6 pairs of peel-colour InDel markers (PS28-PS33) within 3.0–20 Mb upstream and downstream of the peel-colour gene were 55.83–90% in 120 inbred lines. The purity of four commercial hybrids determined using GX1, GX2, PS13, and PS14 was highly consistent with the field results for purity determination. Our results provide important information for genetic linkage map construction, molecular-marker-assisted selective breeding, and purity determination of wax gourd hybrids.

## 1. Introduction

Wax gourd (*Benincasa hispida* (Thunb.) Cogn. (2*n* = 2*x* = 24)), commonly known as white melon, east melon, or pillow melon, is an annual herbaceous climbing species of the Cucurbitaceae family. It originated in China and east India but is currently grown in many tropical, subtropical, and temperate regions of Asia [1], and is widely grown in southern China, Vietnam, Thailand, the Philippines, and other Asian countries. The wax gourd contains small amounts of fat, protein, carbohydrates, and fibre [2]. Wax gourd is a useful raw material suitable for modern agricultural products’ processing and has low planting costs, high yield, a long supply period, and can be stored or transported for long periods. Fruit shape and peel colour significantly vary within the species. The variations in peel colour are due to differences in chlorophyll, carotenoid, and anthocyanin contents [3]. Furthermore, the fruit shape and peel colour of the wax gourd are not only the most important parameters used for evaluating fruit market quality, grading, and pricing, but are also excellent target agronomic traits for genetic improvement of germplasm resources of wax gourd.

A variety of molecular markers have been used to determine hybrid purity [4]; analyse genetic diversity [5]; construct core collections [6]; analyse resistance to fusarium wilt [7], genetic variability for important traits, and kinship [8]; and identify germplasm resources of wax gourd including chieh qua [9]. However, to the best of our knowledge, there are few reports on InDel marker application in wax gourd breeding. InDel marker technology is based on the insertion and deletion of nucleotide sequences at allelic loci in genotypes, which lead to polymorphic variation in sequence length between individuals [10]. To date, InDel markers have been widely developed and utilised in the breeding of a variety of vegetables and fruit such as watermelon [11], cabbage [12], cucumber [13], tomato [14], Chinese cabbage [15], and rape [16]. Previously, our research group fine-mapped the gene that regulates fruit shape (*Bch02G016830*) in the 19.6 Kb region of chromosome 2 [17] and the gene that regulates peel colour (*Bch05G003950*) in the 179 Kb region of chromosome 5 [18]. The fine mapping and cloning of these two genes laid a foundation for the subsequent development of InDel markers for the assisted breeding of wax gourds.

In the present study, based on *Bch02G016830* and *Bch05G003950* genes and resequencing data, we screened InDel mutation loci and developed InDel markers linked to fruit-shape- and peel-colour-related genes. InDel markers were developed to verify the existing wax gourd germplasm resources established by our research group, and we evaluated their potential application in marker-assisted breeding of wax gourd. The developed InDel markers provide a solid theoretical basis for genotypic selection for fruit shape and peel colour, rapid purification of inbred lines, and determination of the degree of hybrid purity between lines with different fruit shapes and peel colour in wax gourd breeding programs.

## 2. Materials and Methods

### 2.1. Experimental Materials

A total of 120 genetically stable and homozygous advanced-generation inbred lines were selected for validation of InDel markers for fruit shape and peel colour, among which 63 were cylindrical and 57 were nearly spherical in fruit shape, while 58 were green and 62 were white in peel colour. Two separate populations of 96 plants for the F_2_ (P_1_: nearly spherical white peel, P_2_: cylindrical green peel) and 440 plants for the F_5_ (P_1_: nearly spherical white peel, P_2_: cylindrical green peel) were prepared for identification of InDel markers for fruit shape and peel colour. Four commercial hybrids, ‘Lvxianzi 2’, ‘Fenxianzi 11’, ‘Chunfeng 818’, and ‘Fenxianzi 1’, were selected to determine hybrid purity with fruit-shape and peel-colour InDel markers. We collected 300 plants of each commercial hybrid (three replicates, 100 plants each). Then two pairs of fruit-shape InDel markers were used for ‘Lvxianzi 2’ and ‘Fenxianzi 11,’ and two pairs of peel-colour InDel markers were used for ‘Chunfeng 818’ and ‘Fenxianzi 1’ for the determination of hybrid purity. The commercial hybrids were provided by Nanning Kenong Seedling Co., Ltd., Nanning, Guangxi, China, and the rest of the plant materials were provided by the Wax Gourd Research Group of the Agricultural College at Guangxi University, Nanning, Guangxi, China.

### 2.2. Experimental Methods

#### 2.2.1. Screening of InDel Marker Loci

The InDel marker loci were screened based on previously obtained resequencing data and gene mapping results for two wax gourd populations (one cylindrical with green peel and the other nearly spherical with white peel) belonging to our research group [17,18]. GatK4 software [19] was used to analyse InDel loci between these two wax gourd populations according to effective resequencing data and the wax gourd reference genome. The InDel loci were selected based on the following criteria: the physical distance was approximately 0.4 Mb upstream and 0.4 Mb downstream from the fruit-shape gene; the physical distance was approximately 20 Mb upstream and 6 Mb downstream from the peel-colour gene (including the gene region), with an insertion and deletion fragment length of ≥3 bp. There were only two SNPS in the fruit-shape gene for which research group members previously designed two pairs of dCAPS markers [18]. The fruit-shape InDel markers developed in this study targeted only the first SNP locus and were used to distinguish between cylindrical (including long and cylindrical) and nearly spherical fruits. The peel-colour InDel markers developed in this study were used to distinguish green-peel and white-peel fruits.

#### 2.2.2. Design and Synthesis of InDel Primers

The selected InDel sites upstream and downstream from the genes of interest, each approximately 200 bp long and nearly 400 bp in total length, were considered for the design of primers using software primer premier 5.0 [20] with the following parameters: primer length ranged from 20 to 28 bp, GC content was 40–60%, annealing temperature was 53–60 °C, and product size containing InDel loci were 100–400 bp. Primer sequences were synthesised by Beijing Qingke Biotechnology Co., Ltd., Nanning, China.

#### 2.2.3. DNA Extraction, PCR Amplification, and 8% Nondenaturing Polyacrylamide Gels Electrophoresis

The CTAB method, with slight modifications, was used to extract total DNA from single plant leaves [21]. DNA was quantified using an ultramicro spectrophotometer (K5800, KAIAO, Beijing, China) and evaluated by electrophoresis on a 1.2% agarose gel. Amplification by PCR was performed to verify the validity of fruit-shape and peel-colour InDel labelling using DNA from 120 inbreds as templates. In turn, the DNA from the parental and isolated populations was used as the template for PCR amplification performed to verify fruit-shape and peel-colour InDel markers in the isolated populations. Additionally, hybrid parental DNA and hybrid DNA were used as templates, and PCR amplification was used to determine hybrid purity of fruit-shape and peel-colour InDel labelling. Purity was estimated according to the following formula [22]:$\;p=\frac{C}{T}\times 100\%,$ where *P* is purity, *C* is the number of samples with both parent characteristic bands, and *T* is the total number of samples detected. The PCR amplification volume was 12 μL and included 2 μL of DNA, 2 μL of primer, 5 μL of Master Mix, and 3 μL of ddH_2_O. PCR amplification was performed using the landing PCR strategy under the following reaction conditions: heat treatment at 94 °C for 5 min; denaturation at 94 °C for 30 s, annealing between 53 and 60 °C for 30 s (annealing temperature was based on the primers), extension at 72 °C for 30 s, 30 cycles; extension at 72 °C for 5 min, followed by preservation in darkness at 16 °C. The PCR amplification products were electrophoresed on 8% nondenaturing polyacrylamide gel electrophoresis at 295 V and a constant pressure of 300 mA for 50 min. After electrophoresis, the PCR products were stained with staining solution (1 g of AgNo_3_ and 1 L of deionised water) for 5–6 min and then cleaned with deionised water before the colour was developed with chromogenic solution (10 g NaOH and 1 L deionised water, dissolved in NaOH and then added to 1.5 mL formaldehyde) for 3 min. After silver staining [23], the results were observed using a gel imaging system.

#### 2.2.4. Construction of Physical Maps of InDel Markers

Based on the name and length information of chromosomes, the physical location of genes, and the physical location of the screened InDel markers of fruit shape and peel colour, the physical map of InDel markers was constructed using MapChart2.32 software (Wageningen University & Research, Wageningen, Netherlands)according to the sequence of genes and InDel markers on chromosomes.

## 3. Results

### 3.1. Physical Map Analysis of InDel Markers

**Figure 1:**
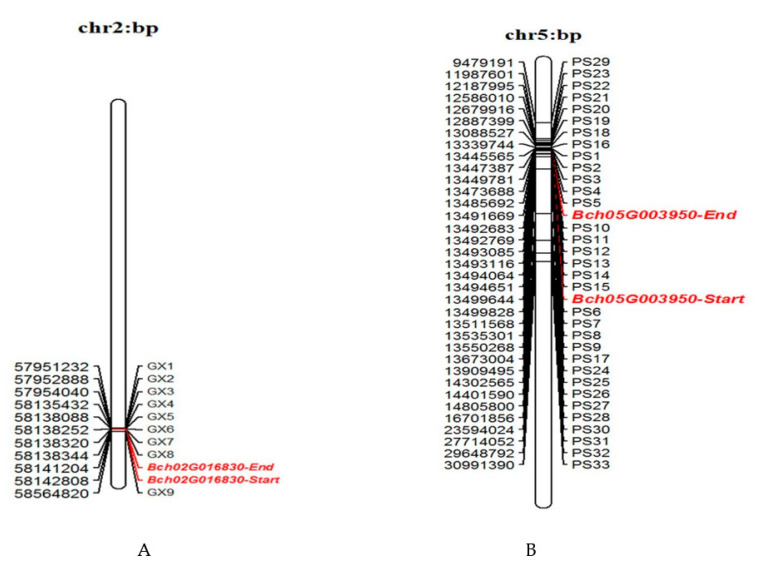
Physical maps of InDel markers linked to fruit-shape (**A**) and peel-colour genes (**B**). Numbers on the left side of the map indicate the physical distance (bp) of the gene and InDel markers. Numbers on the right side are genes and InDel markers.

A total of 9 pairs of fruit-shape and 33 pairs of peel-colour InDel markers with clear amplified bands were identified among 120 inbred lines of wax gourd (Supplementary Tables S1 and S2), and physical maps of fruit-shape and peel-colour InDel markers were constructed (Figure 1). One marker approximately 0.4 Mb upstream and eight markers approximately 0.4 Mb downstream from the gene were detected for the fruit-shape gene. The average physical distance between markers was approximately 6.8176 KB. As for the peel-colour gene, there were 14 markers 20 Mb upstream from the gene, 6 markers in the gene, and 13 markers 6 Mb downstream from the gene. The average physical distance between these markers was 65.1884 KB.

### 3.2. Validation of InDel Markers

According to InDel site selection conditions, 50 pairs of fruit-shape and 80 pairs of peel-colour InDel sites were selected to design primers. A total of nine pairs of fruit-shape InDel markers with clear amplification bands of approximately 0.4 Mb upstream and 0.4 Mb downstream from the fruit-shape gene were obtained (Table 1). The accuracy rate of these InDel markers in the 120 inbred lines ranged from 84.16% to 91.66%. In turn, a total of 33 pairs of peel-colour InDel markers with clear amplification bands within 20 Mb upstream and 6 Mb downstream from the peel-colour gene (including six pairs of markers on the gene) were identified (Table 2). The accuracy rate for the 27 pairs of InDel markers in the 120 inbred lines within about 3.0 Mb upstream and 3.0 Mb downstream (including six pairs of markers on the gene) from the peel-colour gene was 100%. That of six pairs of peel-colour InDel markers within approximately 3.0–20 Mb upstream and downstream from the peel-colour gene varied from 55.83% to 90%. These six pairs of InDel markers began to change and no longer cosegregated at approximately 3.0 Mb upstream and 3.0 Mb downstream from the peel-colour gene. Moreover, the farther the InDel marker from the gene, the lower the degree of linkage.

**Figure 2:**
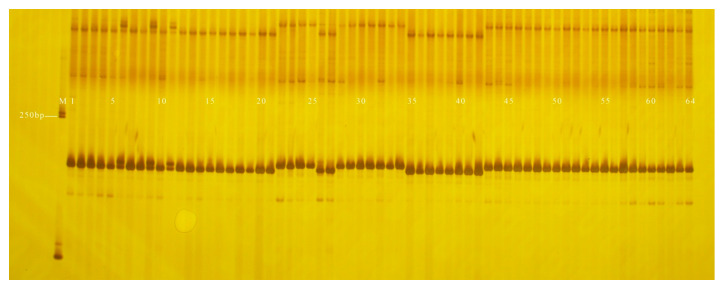
Amplification results of GX1 in some inbred line materials. M: DL2000 DNA Marker; 1–22: cylindrical; 23–25: nearly spherical; 26: cylindrical; 27–32: nearly spherical; 33: cylindrical; 34: nearly spherical; 35: cylindrical; 36–40: nearly spherical; 41–42: cylindrical; 43–64: nearly spherical.

**Figure 3:**
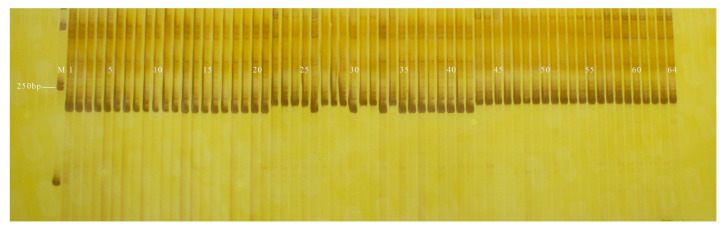
Amplification results of GX7 in some inbred line materials. M: DL2000 DNA Marker; 1–22: cylindrical; 23–25: nearly spherical; 26: cylindrical; 27–32: nearly spherical; 33: cylindrical; 34: nearly spherical; 35: cylindrical; 36–40: nearly spherical; 41–42: cylindrical; 43–64: nearly spherical.

**Figure 4:**
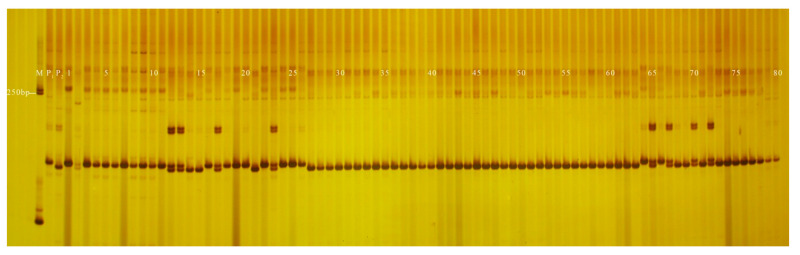
Amplification results of GX9 in some plants isolated from F_5_ populations. M: DL2000 DNA Marker; P_1_: GF-7-1-1 (cylindrical); P_2_: YSB-1-1-2 (nearly spherical); 1–80: F_5_: plants.

**Figure 5:**
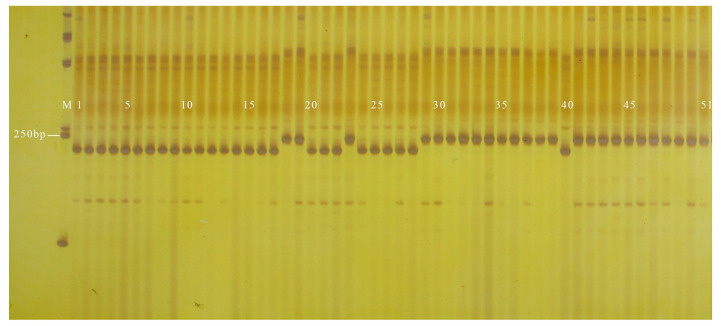
Amplification results of PS13 in some inbred line materials. M: DL2000 DNA. Marker; 1–17: green peel; 18–19: white peel; 20–22: green peel; 23: white peel; 24–28: green peel; 29–39: white peel; 40: green peel; 41–51: white peel.

**Figure 6:**
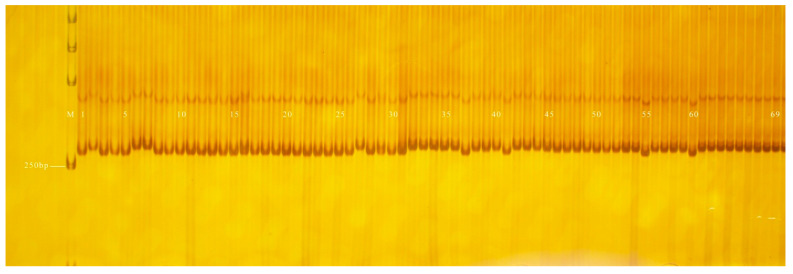
Amplification results of PS28 in some inbred line materials. M: DL2000 DNA Marker; 1–32: green peel; 33–69: white peel.

**Figure 7:**
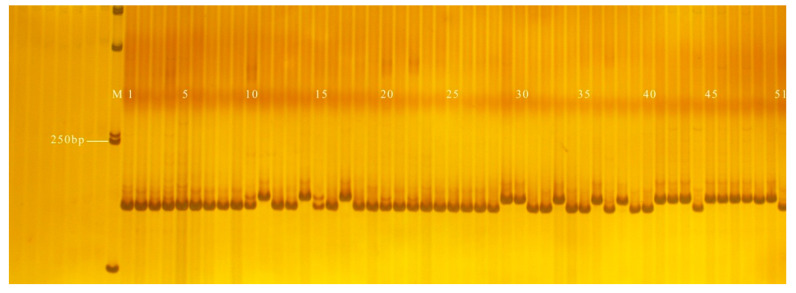
Amplification results of PS30 in some inbred line materials. M: DL2000 DNA Marker; 1–17: green peel; 18–19: white peel; 20–22: green peel; 23: white peel; 24–28: green peel; 29–39: white peel; 40: green peel; 41–51: white peel.

**Figure 8:**
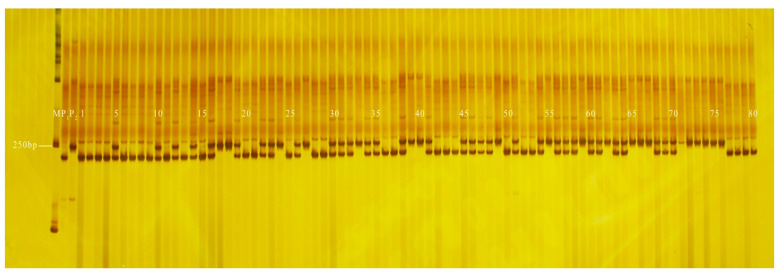
Amplification results of PS13 in some plants isolated from F_5_ populations. M: DL2000 DNA Marker; P_1_: GF-7-1-1 (green peel); P_2_: YSB-1-1-2 (white peel); 1–80: F_5_: plants.

The coincidence rate of GX8 and GX9 in genotyping and field phenotypic identification of the F_2_ (96 plants) and F_5_ (440 plants) segregating populations were 85.42% and 84.09%, respectively; and PS12 and PS13 in genotyping and field phenotypic identification of the F_2_ (96 plants) and F_5_ (440 plants) segregating populations were 95.83% and 95.68%, respectively (Table 3). The fruit-shape and peel-colour InDel markers developed in this study had a higher coincidence rate in the 120 inbred lines but a lower coincidence rate in isolated populations. All 120 inbred lines used in this study were genetically stable, homozygous, advanced generations whose phenotypes were observed over 3 years, while the F_2_ (96 plants) and F_5_ (440 plants) isolated population phenotypes were observed for one year, one plant, and there were environmental errors in the phenotype data (Figure 2, Figure 3, Figure 4, Figure 5, Figure 6, Figure 7 and Figure 8).

### 3.3. Application of InDel Markers in the Determination of Hybrid Purity

**Figure 9:**
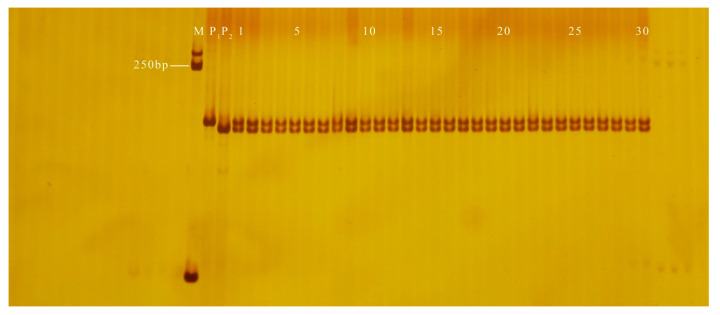
Purity identification of some plants of ‘Lvxianzi 2’ wax gourd by GX1. M: DL2000 DNA Marker; P_1_: GK-3-4-3-2-1-3 (nearly spherical); P_2_: 7-2-1-3-2-2-1 (cylindrical); 1–30: ‘Lvxianzi 2’ wax gourd.

**Figure 10:**
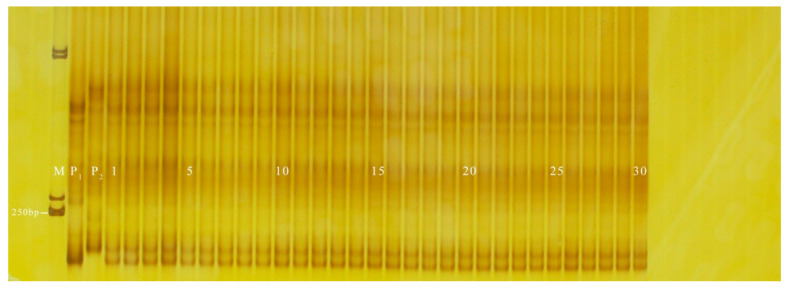
Purity identification of some plants of ‘Fenxianzi 11’ wax gourd by GX2. M: DL2000 DNA Marker; P_1_: ymy-24-1-6-1 (nearly spherical); P_2_: GF-7-1-1 (cylindrical); 1–30: ‘Fenxianzi 11’ wax gourd.

**Figure 11:**
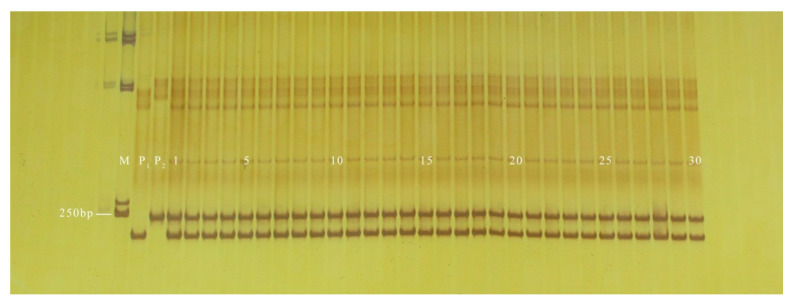
Purity identification of some plants of ‘Chunfeng 818’ wax gourd by PS13. M: DL2000 DNA Marker; P_1_: GK-3-4-3-2-1-3 (green peel); P_2_: T-1-1-1 (white peel); 1–30: ‘Chunfeng 818’ wax gourd.

**Figure 12:**
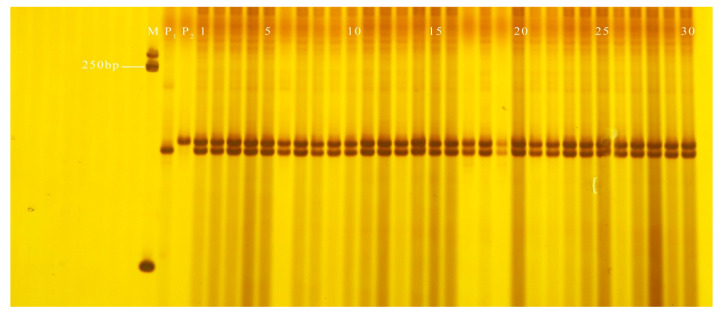
Purity identification of some plants of ‘Fenxianzi 1’ wax gourd by PS14. M: DL2000 DNA Marker; P_1_: YSB-1-1-2 (white peel); P_2_: GF-7-1-1 (green peel); 1–30: ‘Fenxianzi 1’ wax gourd.

Four codominant InDel markers, namely GX1, GX2, PS13, and PS14, were selected to determine the extent of hybrid purity of the commercial hybrids ‘Lvxianzi 2’, ‘Fenxianzi 11’, ‘Chunfeng 818’, and ‘Fenxianzi 1’, respectively (Figure 9, Figure 10, Figure 11 and Figure 12). The results of this test were consistent with those of field determination of the hybrid purity degree (Table 4). Therefore, the InDel markers developed in this study could be used to determine the degree of purity in wax gourd hybrid generations.

## 4. Discussion

Fruit shape and peel colour are important indicators of quality and classification for wax gourd, and both traits are major breeding targets. However, genetically linked fruit-shape- and peel-colour-related InDel markers have not been reported for wax gourd.

Currently, the most widely used PCR-based molecular markers include SSR, KASP, InDel, and CAPS. Under the premise of the known sequence information and SNP site, CAPS markers are subjected to restriction endonuclease digestion of PCR products to generate the target restriction fragment length polymorphism. However, most SNPS are not on the restriction enzyme sites and cannot be transformed into CAPS markers. Therefore, mutated bases are introduced into the flanks of SNP sites by PCR to modify them into restriction endonuclease sites, which generates length polymorphisms of the target fragment. The technique is called derived restriction endonuclease polymorphism markers (dCAPS) analysis [24]. Previously, two pairs of dCAPS markers were reported for two SNPS in the fruit-shape gene by members of our research group [18]. Compared with other markers, dCAPS markers exhibit high genetic stability and wide distribution of loci, but their application is expensive, the operation is complicated, and they have certain limitations with respect to genetic breeding. InDel markers typically show distribution and density within the genome second only to single nucleotide polymorphism markers [25]. Furthermore, InDel-labelled amplification products show a simple and clear band pattern, and their stability and separation are superior to those obtained by SSR labelling. Additionally, InDel markers are simple to operate and do not require much advanced equipment or technology compared with SNP markers. The InDel markers developed in this study can reduce costs and improve efficiency while ensuring high detection accuracy. With the rapid development of high-throughput sequencing technology and reduction in sequencing costs, the use of resequencing data to fine map genes and develop InDel markers closely linked to genes has been extended to several crops, including cucumbers [26], tomatoes [27], *Brassica napus* [28], melons [29], and watermelons [30]. Genes that regulate fruit shape and peel colour have been identified in many crops, and gene-linked InDel markers related to fruit shape have been developed in watermelons [31], cucumbers [32], and melons [33]; those associated with peel colour have been developed in peppers [34], eggplants [35], tomatoes [36], cucumbers [37], chieh qua [3], melons [38], and watermelons [39]. Developing InDel markers linked to genes using InDel-marker-assisted selection breeding can determine phenotypes from genotypes, reduce costs, and improve process efficiency. Furthermore, the technique is not restricted by environmental factors, and the markers can be selected at the seed or seedling stage, thus, shortening the breeding cycle.

In this study, 33 pairs of peel-colour InDel markers were described, 27 pairs of which coseparated in the range of approximately 3.0 Mb upstream and 3.0 Mb downstream (including six pairs of markers on the gene) of the peel-colour gene, with the coincidence rate of 100% in 120 inbred lines. Furthermore, the remaining six pairs of peel-colour InDel markers began to change and no longer cosegregated at approximately 3.0 Mb upstream and 3.0 Mb downstream from the peel-colour gene. The recombination and exchange rates at a physical distance of about 3.0 Mb upstream and 3.0 Mb downstream (including on the gene) were low. This provides great analytical value for the analysis of the hot-spot regions of recombination in the wax gourd genome and future analysis of the degree of regional linkage load. The selected 27 pairs of peel-colour InDel markers could be distinguished among the genotypes of 121 genes within approximately 3.0 Mb upstream and 3.0 Mb downstream from the peel-colour gene.

A total of nine pairs of fruit-shape InDel markers were developed, none of which were on the fruit-shape gene. The rate of coincidence of these markers in the 120 inbred lines ranged from 84.16% to 91.66%, and no coseparating markers were found. The marker closest to the fruit-shape gene was GX8, located 2860 bp downstream from the fruit-shape gene, with a coincidence rate of 90.83% in all 120 inbred lines. This indicated that the interval exchange rate of the region between 0.4 Mb upstream and 0.4 Mb downstream from the fruit-shape gene was higher. The genetic markers developed in this study are more reliable than conventional markers for hybrid purity identification.

## 5. Conclusions

Nine pairs of fruit-shape InDel markers were developed, none of them on the fruit-shape gene. The rate of coincidence of these InDel markers in the 120 inbred lines ranged from 84.16% to 91.66%, and no coseparating markers were found. A total of 33 pairs of peel-colour InDel markers within 20 Mb upstream and 6 Mb downstream from the peel-colour gene (including six pairs of markers on the gene) were identified. The accuracy rate of the 27 pairs of InDel markers in the 120 inbred lines within about 3.0 Mb upstream and 3.0 Mb downstream (including six pairs of markers on the gene) from the peel-colour gene was 100%, while that of 6 pairs of peel-colour InDel markers within approximately 3.0–20 Mb upstream and downstream from the peel-colour gene varied from 55.83% to 90%. These six pairs of InDel markers began to change and no longer cosegregated at approximately 3.0 Mb upstream and 3.0 Mb downstream from the peel-colour gene. Moreover, the farther the InDel marker from the peel-colour gene, the lower the degree of linkage. The fruit-shape and peel-colour InDel markers developed in this study had a higher coincidence rate in the 120 inbred lines, but a lower coincidence rate in isolated populations. The genetic markers developed in this study are more reliable than conventional markers for hybrid purity identification.

The InDel markers developed in the study provide a theoretical basis for genetic linkage map construction, molecular-marker-assisted selective breeding, and purity determination of wax gourd hybrids.

## Figures and Tables

**Table 1:** Coincidence rate of nine pairs of fruit-shape InDel markers in 120 inbred lines.

InDel Markers	Upstream Primer Sequence (5′–3′)	Downstream Primer Sequence (5′–3′)	120 Inbred Lines Coincidence Rate (%)
GX1	GACTTCTTGTTGGGGGTTGA	CATATCATTGACTTGATTGGGC	85.83
GX2	TGTTTTCTTTTTAACAAATGTCGG	GCTGTAATAATCAACCACGGC	86.66
GX3	TCCTTAAAAGTGCTAGTCAATCCA	AAAAAGATTCATCGACATCTCTCC	87.50
GX4	CAGAAGGGGAAAAACCTTGG	TGCTTGAAAAGTTAGACCAAACC	88.33
GX5	GAGTGGTTGTCAAATATTTCGATTT	GAATTTGAAAACAGAAGGAATTAGAG	90.00
GX6	TTGAAGCAGCAACCATACCA	GTGGTAGCCCCCACTTTTCT	89.16
GX7	TTGAAGCAGCAACCATACCA	AGTGGTAGCCCCCACTTTTC	91.66
GX8	TGAAGCAGCAACCATACCAA	GTGGTAGCCCCCACTTTTCT	90.83
GX9	TCATAGATTTGATTTCAGGGCA	CAGTATCATTGAATTCGCACG	84.16

**Table 2:** Coincidence rate of 33 pairs of peel-colour InDel markers in 120 Inbred lines.

InDel Markers	Upstream Primer Sequence (5′–3′)	Downstream Primer Sequence (5′–3′)	120 Inbred Lines Coincidence Rate (%)
PS1	TGACTCCCACAGCAATGAAC	CCTCAATCTTCAAACACAAGCA	100
PS2	CTCTGCAGGCCATCCTCTAT	AATGGAAAATTTCGTGCGTC	100
PS3	GTCGGAGAATCAAGCTTTGC	TTCCATGCATAACCTGGAAAT	100
PS4	TGCCTCCTTTTATCGCATGA	AATACGCCTCCCCCAATTTA	100
PS5	TGCTACGAATTTGTAAATGGTGA	TGGTGGATTTGGAACATCTG	100
PS6	TGTGATGTTTTCTTCAGCCAA	TGTTTGAACGGACAATTACATCTT	100
PS7	TCCCTTTATTAGTTTCTCCCATGA	TTTCTAAAATTGAGTCCAAATCACA	100
PS8	GTGTGCTGATTTGGTTCCCT	AGCCAAGCACGCTTAACATT	100
PS9	GGAAACACCTCGATCTGGAA	CCTCCGTGTTGACTCCCTAA	100
PS10	TGATTAAACCCAATTTGAAAACA	AACAACTGGAGAAATTTGGAGG	100
PS11	CCTCCAAATTTCTCCAGTTG	GCATCTTTCTTAATTACAATGGTTGA	100
PS12	CCAAAAACCAAAAACGAAATG	CCCATCCATTTATTTACGATGA	100
PS13	CCAAAAACCAAAAACGAAATG	TCCCATCCATTTATTTACGATG	100
PS14	TCTATTGCACTGACAAGTGTTTGA	CAAAATTTTTAAAGCTATTGTCCTTC	100
PS15	TCATTACTTCAACCAATCACTCC	GCAACAAGGAATTCAGCCAT	100
PS16	TTACTTTTCCTCATCCAATTTCTAA	GCATTGCACGTGTTATATAAAGTCT	100
PS17	CCGATTCATGTGACCTTGAC	GCAGACAATCCACAAACCAC	100
PS18	CAAAACCGTTACTAAACCAGACC	TGAAGGTGTCTGTGCTTTTCT	100
PS19	GACATGGCTAAGTCAAGGGC	TGACGAAAAAGAAAATTTTGGAA	100
PS20	TGGCAGAACCCAAATTTATTG	AAGGGGAGGGGATTGATAAAG	100
PS21	TTCTCTACGCTGAGCCGTTT	GGGGAGGCAAACCAAATAAT	100
PS22	TGGTGTGAAGGAAGGTGGTT	TTTTGGGGCAAAGTCTAACCT	100
PS23	AAAAGAAATAACTCTTGAAAATGTTTG	CCAATTGCCTTTTGCATTTT	100
PS24	CCACCATCTGTTAACTGCCA	GCATGCACATGCTTTCTTGT	100
PS25	AACATCCAAAATTTGCACCA	CCTCATCTTCCAACAACTGTCA	100
PS26	GGTTAAAAGATAAGCGGTTTGATT	AACCTCCCTCCACTCCCTTA	100
PS27	TTCCTAGCCAGTTTGTCATTCA	AAAGCCATCATCTCTATTCCTCA	100
PS28	AGGGTGAAATCCCGAAGAAG	TGATAGTTACCCCCGTTCGT	90.00
PS29	AAAACCAAGCCGACTTTTGA	TTTGGTAACCATTTCATCTTTGG	86.66
PS30	GCAATTTCAGACGGTGGTTT	TCCCTTGCCTTTCTGCTTTA	65.00
PS31	TCAAAAGGCTCAAAACCCAC	TGTTGCTGCATTTCCATTGT	60.00
PS32	CATGGTCAACGATGTGGAAG	AGAGTGGGTGGAAAGCGTTA	56.66
PS33	GTGCAAGCTTATGCCATTGA	AAAGGTCAAACAAATGAGTGTTCA	55.83

**Table 3:** Coincidence rate of GX8, GX9, PS12, and PS13 in F_2_ (96 plants) and F_5_ (440 plants).

InDel Markers	Coincidence Rate of F_2_ (96 Plants) (%)	Coincidence Rate of F_5_ (440 Plants) (%)
GX8	85.42	
GX9		84.09
PS12	95.83	
PS13		95.68

**Table 4:** Comparison of field grow-out trials (GOT) and InDel molecular marker purity test for wax gourd.

Hybrid	InDel Assessment Average (%)	GOT Assessment Average (%)	Purity Deviation Range (%)
‘Lvxianzi 2’ wax gourd	98.3	97.0	1.3
‘Fenxianzi 11’ wax gourd	100.0	100.0	0.0
‘Chunfeng 818’ wax gourd	99.0	98.3	0.7
‘Fenxianzi 1’ wax gourd	98.0	98.6	0.6

Note: Student T test: T = 0.461.

## Data Availability

The data presented in this study are available in this article.

## Supplementary Materials

The following supporting information can be downloaded at: https://www.mdpi.com/article/10.3390/genes13091567/s1, Table S1. Physical map information of fruit-shape InDel markers; Table S2. Physical map information of peel-colour InDel markers.
