# Supplementary material for: Development and Application of InDel Markers Linked to Fruit-Shape and Peel-Colour Genes in Wax Gourd
Source: Genes (Basel). 2022 Aug 31;13(9):1567. doi: 10.3390/genes13091567 (PMC9498789; doi:10.3390/genes13091567)
Supplement: Supplementary file 1 [file genes-13-01567-s001.zip › genes-1867755-supplementary.pdf]

Supplementary Materials:

Table S1. Physical map information of fruit-shape InDel markers.

Gene/InDel Markers	Chromosome	Physical Location (bp)	Length of Chromosome (bp)
Bch02G016830	Chromosome 2	58141204-58142808	68019380
GX1	Chromosome 2	57951232	
GX2	Chromosome 2	57952888	
GX3	Chromosome 2	57954040	
GX4	Chromosome 2	58135432	
GX5	Chromosome 2	58138088	
GX6	Chromosome 2	58138252	
GX7	Chromosome 2	58138320	
GX8	Chromosome 2	58138344	
Bch02G016830-End	Chromosome 2	58141204	
Bch02G016830-Start	Chromosome 2	58142808	
GX9	Chromosome 2	58564820	

Table S2. Physical map information of peel-colour InDel markers

Gene/InDel Markers	Chromosome	Physical Location (bp)	Length of Chromosome (bp)
Bch05G003950	Chromosome 5	13491669-13499644	68378982
PS29	Chromosome 5	9479191	
PS23	Chromosome 5	11987601	
PS22	Chromosome 5	12187995	
PS21	Chromosome 5	12586010	
PS20	Chromosome 5	12679916	
PS19	Chromosome 5	12887399	
PS18	Chromosome 5	13088527	
PS16	Chromosome 5	13339744	
PS1	Chromosome 5	13445565	
PS2	Chromosome 5	13447387	
PS3	Chromosome 5	13449781	
PS4	Chromosome 5	13473688	
PS5	Chromosome 5	13485692	
Bch05G003950-End	Chromosome 5	13491669	
PS10	Chromosome 5	13492683	
PS11	Chromosome 5	13492769	
PS12	Chromosome 5	13493085	
PS13	Chromosome 5	13493116	
PS14	Chromosome 5	13494064	
PS15	Chromosome 5	13494651	
Bch05G003950-Start	Chromosome 5	13499644	
PS6	Chromosome 5	13499828	
PS7	Chromosome 5	13511568	
PS8	Chromosome 5	13535301	
PS9	Chromosome 5	13550268	
PS17	Chromosome 5	13673004	
PS24	Chromosome 5	13909495	
PS25	Chromosome 5	14302565	
PS26	Chromosome 5	14401590	
PS27	Chromosome 5	14805800	
PS28	Chromosome 5	16701856	
PS30	Chromosome 5	23594024	
PS31	Chromosome 5	27714052	
PS32	Chromosome 5	29648792	
PS33	Chromosome 5	30991390	
